# Grynfeltt Hernia: A Rare Case With Serious Complications

**DOI:** 10.7759/cureus.96009

**Published:** 2025-11-03

**Authors:** Aymen Abdelmounaime Mehdaoui, Moath Sarhan, Khedidja Belkharroubi, Remouche Hafid, Amina Guennich

**Affiliations:** 1 Department of General Surgery, University Hospital of Oran, Oran, DZA

**Keywords:** bowel obstruction, grynfeltt-lesshaft hernia, incarcerated lumbar hernia, recurrence, superior lumbar triangle hernia

## Abstract

Grynfeltt-Lesshaft hernia is a rare type of abdominal hernia. The diagnosis remains challenging, particularly in urgent clinical scenarios. The choice of a surgical technique remains controversial, as each approach carries specific advantages and limitations. We present a case of an incarcerated lumbar hernia through the superior lumbar triangle with a large focal defect identified on diagnostic imaging, which led to an early recurrence and a sequence of complications, including bowel perforation, colonic necrosis, and abscess formation with fistulation. This case highlights the complexity of surgical decision-making in emergency presentations and the importance of selecting the appropriate method for primary repair of rare abdominal defects.

## Introduction

Lumbar hernias manifest as abnormal protrusions of intra-abdominal content through two main areas, namely, the superior lumbar triangle (Grynfeltt-Lesshaft triangle) and the inferior lumbar triangle (Petit triangle) [1]. Grynfeltt hernia is a defect of the superior lumbar triangle, which is formed medially by the erector spinae muscle group, laterally by the internal oblique muscle, and superiorly by the 12th rib. The transversalis muscle’s aponeurosis forms the inferior boundary of this triangle, with the latissimus dorsi muscle serving as its roof [2]. These defects constitute the rarest type of abdominal wall hernia, accounting for only 2% of all cases, with approximately 300 documented reports worldwide [3-7].

Based on etiology, these hernias are classified as congenital, resulting from developmental defects, which account for approximately 20% of cases. The remaining (80%) are acquired [5,8,9], further subdivided into primary idiopathic hernias (55%) and secondary hernias (25%). The latter typically result from trauma or prior surgical intervention [8].

The pathophysiology involves failure at three anatomical weak points: first, below the 12th rib, where the transversalis fascia lacks external oblique coverage; second, at the penetration site of the 12th intercostal neurovascular pedicle; and third, between the rib’s inferior edge and the ligament of Henle [2,4,5].

Diagnosis is challenging due to non-specific symptoms of lumbar pain and reducible masses that can enlarge with straining. Differential diagnosis includes soft-tissue tumors and inflammatory tumefactions, which lack the characteristic positional changes and cough-impulse sign [7-11]. While clinical examination by palpation and the Valsalva maneuver is essential, CT is the ideal imaging modality for anatomical assessment and the identification of fascial defects [2,7,10].

Definitive treatment involves surgical repair, performed via open or laparoscopic techniques, using synthetic mesh, often combined with muscle flaps, depending on the defect’s characteristics [10]. Given the condition’s rarity, the average surgeon may encounter only one case in their career [2,4,7].

Here, we describe a rare case of a complicated Grynfeltt hernia resulting in segmental colonic necrosis, providing valuable insights into the limited literature on the severe complications of lumbar hernias.

## Case presentation

A 67-year-old man with a three-year history of an intermittent palpable mass in the left lumbar region presented to the emergency department with a three-day history of absolute constipation associated with nausea and vomiting. His past medical history was significant for a prosthetic right eye from childhood trauma, with no other medical comorbidities.

**Figure 1:**
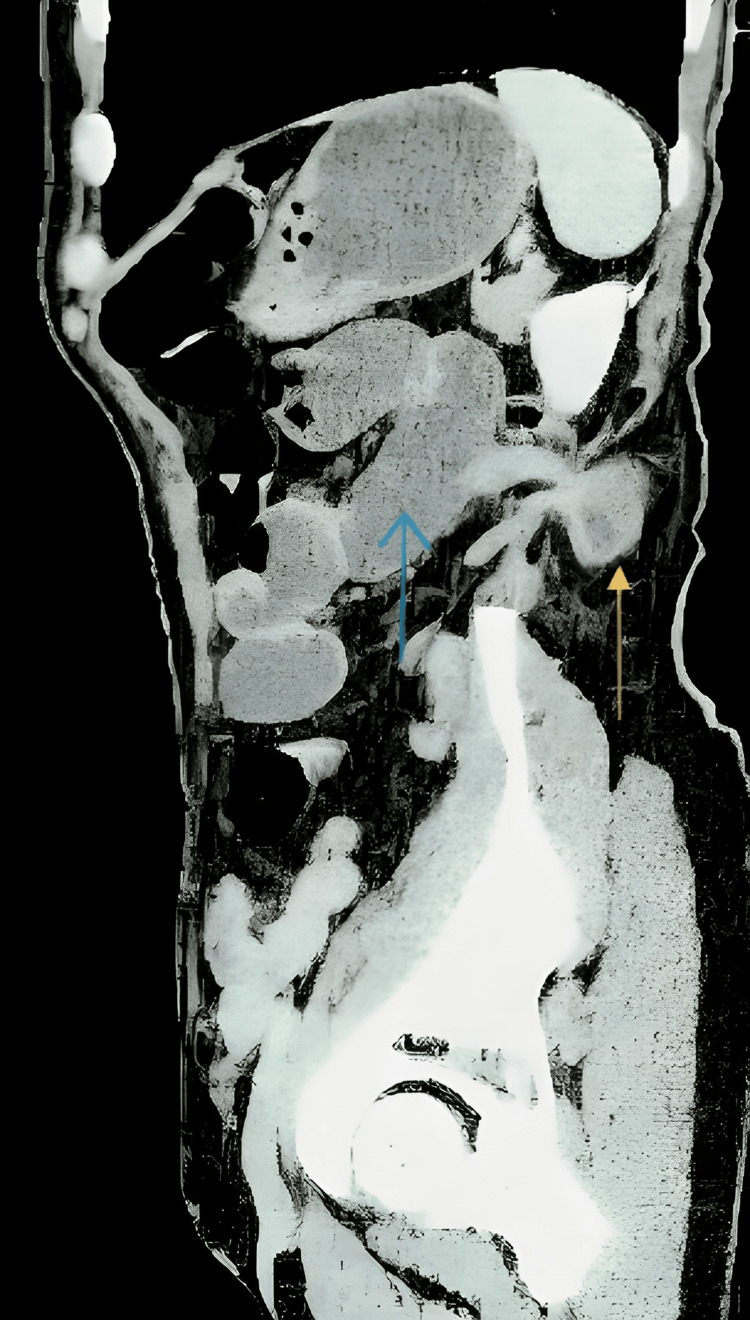
Sagittal CT scan section showing an incarcerated small bowel loop herniating through a superior lumbar fascial defect. Dilated bowel loop (yellow arrow) herniates through a 17 mm posterior lumbar fascial defect. Proximal bowel dilatation (blue arrow) indicates mechanical obstruction consistent with a Grynfeltt-Lesshaft hernia.

**Figure 2:**
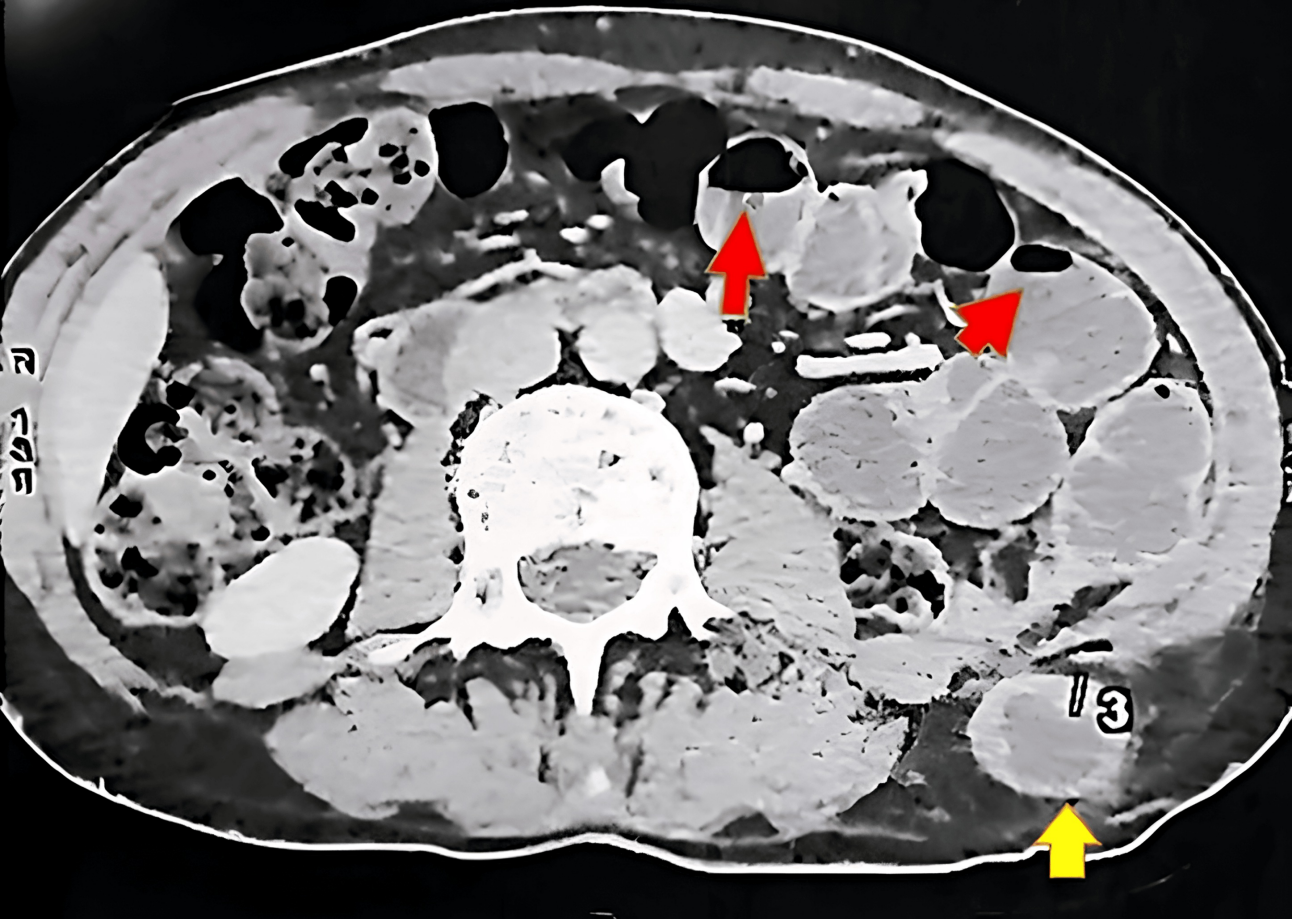
Axial CT scan demonstrating air-fluid levels due to bowel obstruction secondary to an incarcerated lumbar hernia. Dilated small bowel loops with air-fluid levels (red arrows) indicate mechanical obstruction. The incarcerated bowel segment in the left lumbar hernia (yellow arrow) shows a 30 mm loop dilatation with 8 mm wall thickening (3) from compromised perfusion.

Physical examination revealed abdominal distension with tympany on percussion and a left fixed, mildly tender, irreducible lumbar mass that did not expand with coughing. Moderate atrophy of the paraspinal muscles was also noted. An abdominal CT scan revealed a 17 mm focal defect in the posterolateral musculo-fascial layer, accompanied by an incarcerated loop of the small bowel within the superior lumbar triangle (Figure 1), resulting in a complete bowel obstruction (Figure 2).

The patient was immediately admitted for emergency laparotomy. Through a total abdominal midline incision, exploration identified an incarcerated jejunal loop through the defect, located 40 cm distal to the ligament of Treitz. The loop was manually reduced back into the peritoneal cavity, appeared viable with the rest of the intestinal segment, and had minimal serosal tearing, which was repaired using interrupted 3/0 non-absorbable sutures. The posterior parietal peritoneum was closed using continuous 3/0 absorbable sutures. The underlying musculo-fascial defect of the lumbar hernia was not addressed, with definitive hernia repair planned after complete recovery from the emergency surgery.

**Figure 3:**
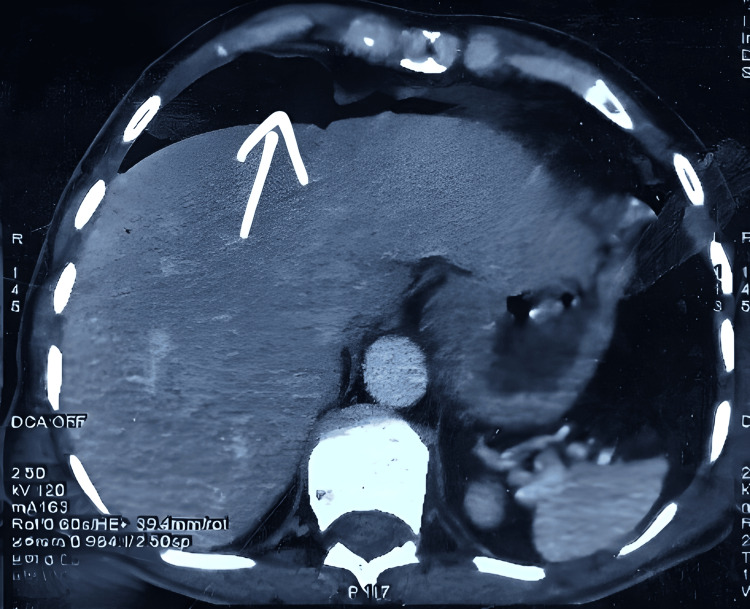
Axial CT scan showing pneumoperitoneum. Significant pneumoperitoneum with free air (white arrow) indicates bowel perforation, appearing as hypodense areas throughout the peritoneal cavity.

**Figure 4:**
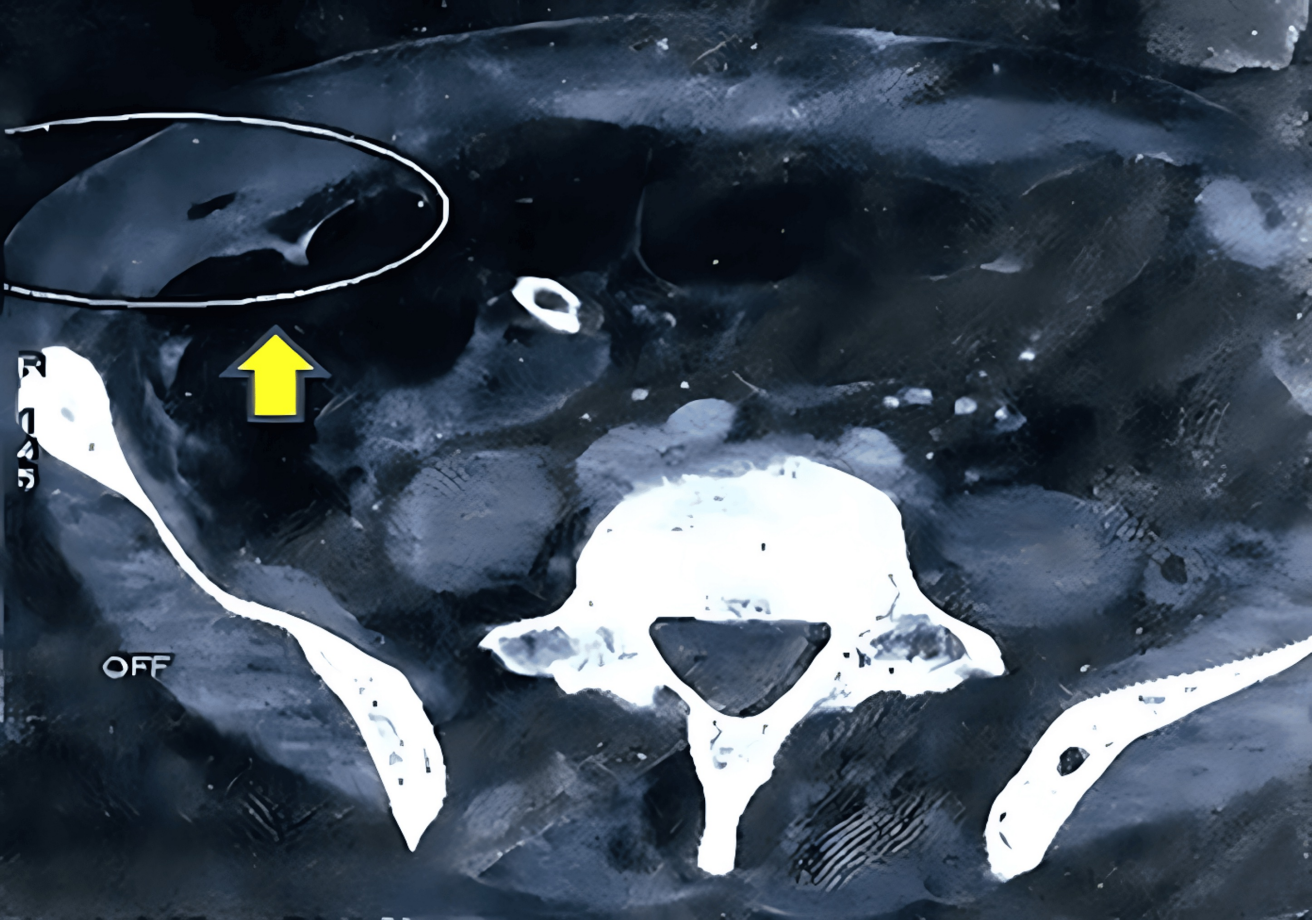
Axial CT scan section showing a right-sided parietal wall abscess with localized peritoneal reaction. Rim-enhancing fluid collection in the right parietal wall (yellow arrow, circled) is consistent with an abscess. Surrounding soft-tissue stranding indicates a localized inflammatory reaction.

Three days after the operation, the patient experienced severe abdominal pain. The physical examination revealed signs consistent with peritonitis. The CT scan revealed pneumoperitoneum (Figure 3) and a right-sided parietal wall abscess with localized peritoneal reaction (Figure 4), necessitating immediate surgical re-exploration. Laboratory investigations revealed leukocytosis consistent with the septic process and mildly elevated mean corpuscular volume, signifying macrocytic anemia (Table 1).

**Table 1:** Preoperative complete blood count before emergency laparotomic exploration (second surgery). Laboratory results obtained on postoperative day three demonstrated leukocytosis with a left shift (elevated neutrophils) and macrocytic anemia, consistent with acute sepsis.

Test	Result	Unit	Reference range
White blood cell count	17.85	10³/µL	4.00–10.00
Neutrophils	16.04	10³/µL	2.00–7.00
Hemoglobin	11.4	g/dL	13.0–16.0
Mean corpuscular volume	104.6	fL	80.0–100.0
Mean corpuscular hemoglobin	35.6	pg	27.0–34.0
Platelets	460	10³/µL	150–450

The abdomen was accessed through the previous midline incision. Surgical exploration revealed recurrence of herniation through the original defect, now containing a strangulated left colonic flexure with its omentum. The affected colon was non-viable with advanced ischemic necrosis and perforation with fecal contamination of the peritoneal cavity. These findings were consistent with the source of the pneumoperitoneum and septic peritonitis.

We proceeded with an urgent segmental left colonic resection of the necrotic portion using a GIA-80 stapler with reinforcement using interrupted 3/0 absorbable sutures. Viable, well-perfused bowel margins were confirmed. A two-layer, hand-sewn, latero-lateral, colo-colic anastomosis was created with 3/0 absorbable sutures. The necrotic omentum was excised, followed by extensive peritoneal lavage with warm saline to address the fecal peritonitis. The procedure was completed with primary fascial repair using 1/0 polypropylene suture in a cruciate (figure-of-eight) pattern. Histopathological examination confirmed ischemic colonic necrosis with clear margins. The patient was discharged on postoperative day five (POD 5).

**Figure 5:**
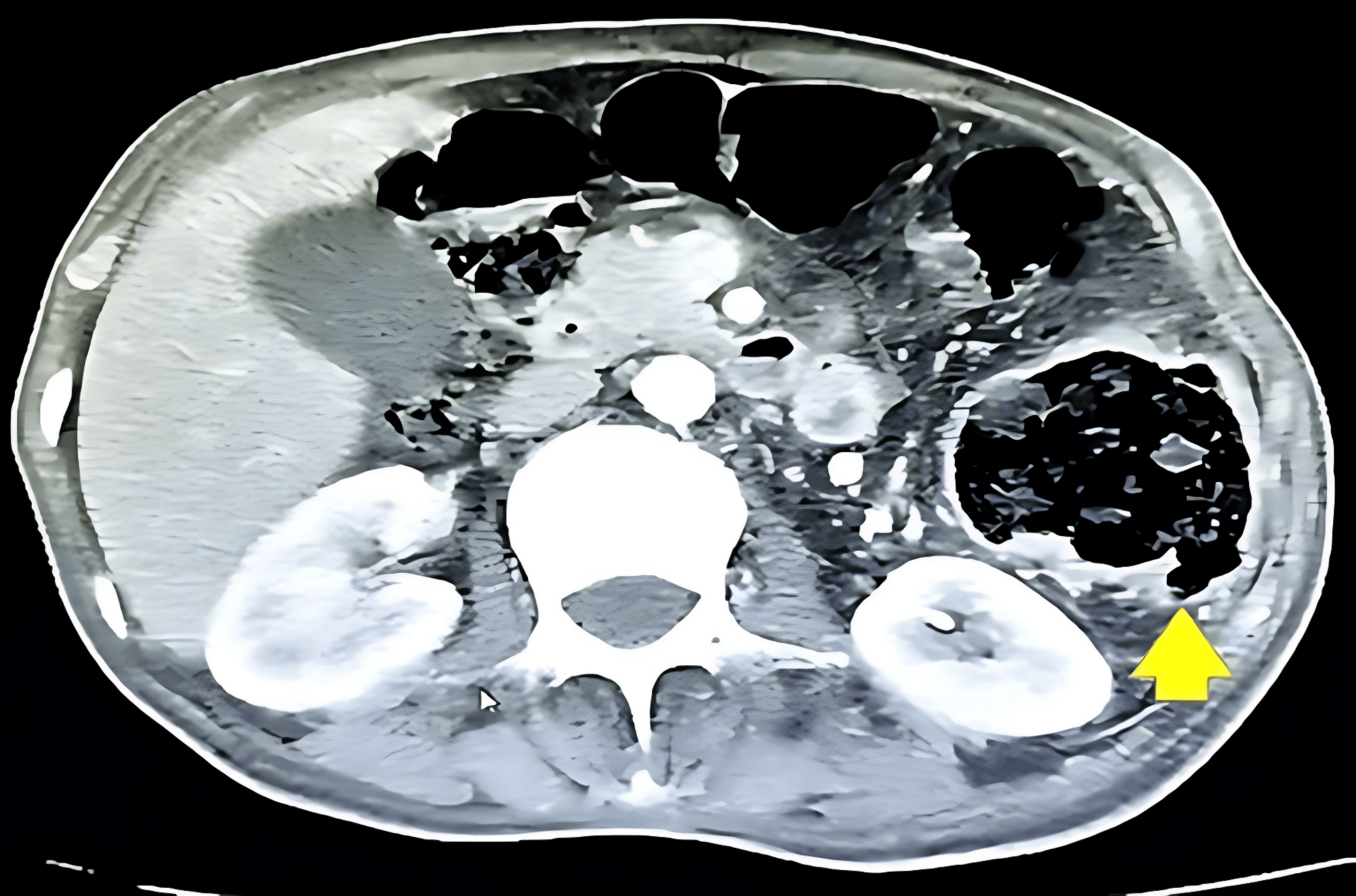
Axial CT scan showing a large lumbar abscess. Large hypodense fluid collection (yellow arrow) extends from the left lower quadrant to the posterior lumbar region, consistent with a complex abscess measuring approximately 20 mm in thickness and 200 mm in length.

Five weeks later, the patient presented with abdominal pain and yellowish discharge from the skin. A CT scan revealed a large abscess extending from the left lower quadrant to the posterior lumbar region (20 mm × 200 mm) (Figure 5), with fistulation to the skin. The patient was readmitted to the operating room for abscess debridement.

Abscess specimen analysis showed a weakly positive acid-fast bacillus, while histopathology revealed a pyogenic granuloma without evidence of malignancy. The postoperative incisional wound was managed with open packing and daily dressing changes. Due to concern for active tuberculosis, a conservative approach with a protective colostomy was planned.

Intraoperative exploration revealed anastomotic dehiscence with a small leak. The anastomosis was taken down with assessment of bowel viability. A terminal transverse colostomy was created through a separate left upper quadrant incision, with the distal colonic stump closed and returned to the peritoneal cavity in a Hartmann configuration. Peritoneal lavage was performed. Operative cultures yielded *Escherichia coli*.

Subsequent tuberculosis workup (QuantiFERON, intradermal test, aerobic cultures) returned negative. Following specialist consultation, anti-tubercular treatment was deemed unnecessary, and broad-spectrum antibiotics were continued. The patient was discharged on POD 8 with clinical improvement.

Colonic continuity restoration was performed 17 months later through the previous midline incision. Following adhesiolysis, the transverse colostomy was mobilized and taken down; both bowel ends were trimmed to healthy, well-vascularized tissue. A two-layer, hand-sewn, end-to-end, colo-colic anastomosis was created using 3/0 absorbable sutures. Mesenteric defects were approximated, and fascial closure was performed with non-absorbable sutures. The patient recovered without complications, with bowel function returning on POD 3 and discharge on POD 5. Long-term follow-up demonstrated no anastomotic complications or hernia recurrence (Table 2).

**Table 2:** Timeline of clinical events and interventions. A chronological summary of operative interventions, key findings, and outcomes in the presented Grynfeltt hernia case. The table provides exact dates, corresponding postoperative days (referenced from the initial laparotomy on March 4, 2023), and the discharge dates to illustrate the complexity of the clinical course. Intervals (e.g., five weeks, one week) are calculated from the preceding discharge dates. POD: postoperative day; SBO: small bowel obstruction

Timeline	Key findings/Interventions	Discharge dates and actions
Day 0 (03/05/2023) POD 0	Findings: irreducible hernia, complete SBO, a 17 mm fascial defect with jejunal incarceration. Intervention: laparotomy; reduced incarcerated jejunum, repaired its serosal tear. Fascial defect repair was not attempted	POD 1: transferred from the emergency department to the general surgery department
Day 3 (03/08/2023) POD 3	Findings: peritonitis, pneumoperitoneum, abscess. Necrotic left colonic flexure found. Intervention: laparotomic re-exploration; left colectomy, side-to-side anastomosis, definitive fascial repair with figure-of-eight sutures	03/13/2023
5 weeks later (04/17/2023) POD 43	Findings: large abscess with cutaneous fistulation. Intervention: surgical abscess debridement	Remained as an inpatient
1 week later (04/25/2023) POD 51	Decision: conservative approach with a protective colostomy. Findings: anastomotic leak. Intervention: takedown of anastomosis, creation of terminal transverse colostomy, broad-spectrum antibiotics continued. Abscess resolution	05/03/2023
16 months later (09/25/2024)	Intervention: colostomy reversal. Outcome: uneventful recovery, successful restoration of colonic continuity	09/30/2024

## Discussion

Grynfeltt-Lesshaft hernia is the rarest form of abdominal hernia, occurring in the lumbar region, which is divided into the following two areas: at the top of the 12th rib (Grynfeltt-Lesshaft), and below the iliac crest line (Jean Louis Petit) [5,6].

Lumbar hernia occurs as an acquired condition in 80% of cases. It is classified as either primary, which is associated with obesity, muscular atrophy, and chronic debilitating diseases (e.g., HIV) [4,6], or traumatic, which occurs after lumbar trauma, direct contusion, surgery (e.g., flank surgery), or infections (e.g., hepatic abscesses, lumbar tuberculosis) [2,4-6]. In contrast, 20% of cases are congenital and often linked with lumbar vertebral defects, meningocele, and neurofibromatosis [6]. In this case, the patient developed an acquired lumbar hernia, likely due to age-related muscle atrophy and decreased body mass.

Regarding clinical presentation, the typical presentation of lumbar hernias is a reducible swelling with cough impulse; our patient had an atypical, irreducible hernia without expansion or cough response. Most patients with lumbar hernia are diagnosed in outpatient settings, and about 9% present urgently with incarceration or strangulation. Moreover, 25% of cases have no auscultatory signs, as demonstrated in our case [4,6]. Further, 91% of cases present as chronic and are less severe forms [6-8,12]. According to Moreno-Egea’s classification, the present case meets Type A criteria (size <5 cm, location: superior, etiology: spontaneous) and Type B criteria (visceral contents, muscle atrophy, and recurrence). We classified it as Type B, given the visceral incarceration and muscle atrophy, which are more clinically significant than size parameters alone (Table 3) [7].

**Table 3:** Clinical classification of lumbar hernias with the suggested surgical approaches. Types A, B, and C represent true fascial defects. Type D (pseudohernia) indicates severe muscular atrophy without a fascial defect. The presence of at least two criteria is necessary for defining a type.

Characteristic	A	B	C	D (pseudohernia)
Size, cm	<5	5–15	>15	-
Location	Superior	Inferior	Diffuse	-
Contents	Extraperitoneal fat	Visceral	Visceral	-
Etiology	Spontaneous	Incisional	Traumatic	-
Muscular atrophy	No (minor)	Mild	Severe	Severe
Recurrence	No	Yes (open)	Yes (laparoscopy)	-
Surgical approach	Open approach, extraperitoneal laparoscopy, total extraperitoneal laparoscopy	Intraperitoneal laparoscopy	Open approach	Open approach (double mesh)

CT scan or MRI are reported as the gold standard examinations for lumbar hernias, providing detailed evaluation of the hernial defect, contents, surrounding tissues, and guiding the type of surgical approaches [6]. Lumbar hernia in the elderly typically presents as large and prominent, making clinical diagnosis straightforward. In contrast, lumbar hernia in younger patients is often asymptomatic without a relevant history. The wide differential diagnosis (including cold abscesses, lipomas, hematomas, fibromas, or renal tumors) makes diagnosing lumbar hernia in younger patients challenging [6,8,9].

Surgical repair of lumbar hernias is considered the ideal choice. Various techniques have been described, including no repair, primary suture, muscle flap reconstructions, and prosthetic mesh reinforcement [13]. The most common methods are open repair and the laparoscopic approach. The open approach remains the most widely used, due to its safety, effectiveness, and economic advantages [3,10].

During the initial emergency intervention, the surgeon prioritized bowel decompression over hernia repair, focusing on relieving the acute intestinal obstruction. In retrospect, this approach was incomplete. Merely liberating the incarcerated portion, without addressing the fascial defect, particularly in an elderly patient with tissue atrophy, can result in early recurrence and serious complications such as strangulation, which may potentially require bowel resection [3,12].

In contrast, prosthetic mesh repair has proven effective in addressing large defects [8]. Moreno-Egea et al. have stated that the repair must be performed under proper tension in cases with significant muscle atrophy and/or major deformity to achieve aesthetic and functional outcomes [7]. Our experience and literature support the use of a mesh or, preferably, the sandwich technique, as described by Sahoo et al [14]. The latter involves placing an underlay polypropylene mesh extraperitoneally to cover the defect and an overlay mesh to sandwich the muscle layers [14]. This method prevents recurrence, lowers infection rates, and ensures optimal anatomical wall reconstruction [11,14].

The laparoscopic approach offers many advantages, such as minimal invasiveness, excellent defect visualization, lower infection rates, reduced pain, and shorter hospital stays [3,7,12]. However, bowel strangulation required open repair in our case. The choice between approaches should be guided by factors such as age, tissue quality, defect size, and clinical urgency [8].

## Conclusions

Spontaneous lumbar hernias are rare clinical entities that often present a diagnostic challenge, particularly in emergency settings. Complete bowel obstruction from an existing lumbar hernia can be an indicator of its presence. Due to the risk of incarceration, strangulation, and subsequent life-threatening complications such as bowel necrosis, prudent diagnostic and therapeutic management, with a thorough understanding of the regional anatomy of the defect, is crucial. Guided by current classifications and our clinical experience, we advocate for an open approach with mesh reinforcement to achieve a tension-free repair. In emergency presentations, priority must be given to life-threatening visceral complications; however, the fascial defect must be addressed with at least primary closure to prevent early recurrence and its severe consequences.
